# Deep learning-based algorithm improves radiologists’ performance in lung cancer bone metastases detection on computed tomography

**DOI:** 10.3389/fonc.2023.1125637

**Published:** 2023-02-08

**Authors:** Tongtong Huo, Yi Xie, Ying Fang, Ziyi Wang, Pengran Liu, Yuyu Duan, Jiayao Zhang, Honglin Wang, Mingdi Xue, Songxiang Liu, Zhewei Ye

**Affiliations:** ^1^ Department of Orthopedics, Union Hospital, Tongji Medical College, Huazhong University of Science and Technology, Wuhan, China; ^2^ Research Institute of Imaging, National Key Laboratory of Multi-Spectral Information Processing Technology, Huazhong University of Science and Technology, Wuhan, China; ^3^ Department of Radiology, Union Hospital, Tongji Medical College, Huazhong University of Science and Technology, Wuhan, China

**Keywords:** artificial intelligence, deep learning, deep convolutional neural network, lung cancer bone metastases, computer-aided diagnosis

## Abstract

**Purpose:**

To develop and assess a deep convolutional neural network (DCNN) model for the automatic detection of bone metastases from lung cancer on computed tomography (CT)

**Methods:**

In this retrospective study, CT scans acquired from a single institution from June 2012 to May 2022 were included. In total, 126 patients were assigned to a training cohort (n = 76), a validation cohort (n = 12), and a testing cohort (n = 38). We trained and developed a DCNN model based on positive scans with bone metastases and negative scans without bone metastases to detect and segment the bone metastases of lung cancer on CT. We evaluated the clinical efficacy of the DCNN model in an observer study with five board-certified radiologists and three junior radiologists. The receiver operator characteristic curve was used to assess the sensitivity and false positives of the detection performance; the intersection-over-union and dice coefficient were used to evaluate the segmentation performance of predicted lung cancer bone metastases.

**Results:**

The DCNN model achieved a detection sensitivity of 0.894, with 5.24 average false positives per case, and a segmentation dice coefficient of 0.856 in the testing cohort. Through the radiologists-DCNN model collaboration, the detection accuracy of the three junior radiologists improved from 0.617 to 0.879 and the sensitivity from 0.680 to 0.902. Furthermore, the mean interpretation time per case of the junior radiologists was reduced by 228 s (p = 0.045).

**Conclusions:**

The proposed DCNN model for automatic lung cancer bone metastases detection can improve diagnostic efficiency and reduce the diagnosis time and workload of junior radiologists.

## 1 Introduction

Lung cancer (LC) is the main cause of cancer-related deaths globally (1). Approximately 1.5 million people are diagnosed with LC every year, with 1.3 million deaths (2). Furthermore, bone is the most common and the initial site of metastases from LC (3). Approximately 30%–70% of bone metastases are associated with LC, and 20%–30% of patients with LC already have bone metastases upon initial diagnosis (4). LC is often asymptomatic at the initial stage. Therefore, patients possibly already have metastases at diagnosis (5, 6). Although bone metastases may progress to pathologic fracture and/or nerve and spinal cord compression; some patients have no painful symptoms at the time of detection (7, 8). Once a tumor metastasizes to the bone, it is practically incurable and has a high mortality rate (9). Therefore, early detection of bone metastasis is important for decreasing morbidity as well as for disease staging, outcome prediction, and treatment planning (10).

Imaging is an important part of the management of bone metastasis (11–13). Computed tomography (CT) has the advantages of good anatomical resolution, soft-tissue contrast, and detailed morphology (14, 15). It also facilitates simultaneous evaluation of the primary and metastatic lesions (12, 16). The most important advantage of CT is the relatively low cost, which is very patient-friendly (17). Thus, in the clinical setting, CT is the most commonly used imaging for the diagnosis of primary cancer and whole-body staging when bone metastases are suspected (17, 18). The measurements of all metastatic lesions are time-consuming, especially, if multiple metastases are present. The heavy workload of image evaluation can be tiresome for radiologists, thus increasing the risk of missing lesions and leading to decreased sensitivity (19). Therefore, automated analysis of CT images is ideal for assisting radiologists in the accurate diagnosis of bone metastasis from LC.

Deep learning has been identified as a key sector in which artificial intelligence could streamline pathways, acting as a triage or screening service, decision aid, or second-reader support for radiologists (20). By now, artificial intelligence with deep convolutional neural network (DCNN) has been exploited to develop automated diagnosis and classification of cancer, including prostate cancer (21, 22), pancreatic cancer (23), gastric cancer (24, 25), breast cancer (26, 27), and LC (28–30). Furthermore, there has been a line of research on DCNN-based automated classification of CT images for the detection of metastasis caused by various primary tumors including gastric cancer (30), breast cancer (31), LC (32), and thyroid cancer (33). There is emerging evidence suggesting that DCNN could also be used to extract information from bone scan images for the automatic detection of LCBM (34, 35).

In this study, we developed a DCNN model that automated detecting LC bone metastases (LCBM) on CT and validated the model internally and externally. We also compared the DCNN model with five radiologists and explored whether it could enhance the diagnostic accuracy of junior radiologists.

## 2 Materials and methods

### 2.1 Patients

**Figure 1:**
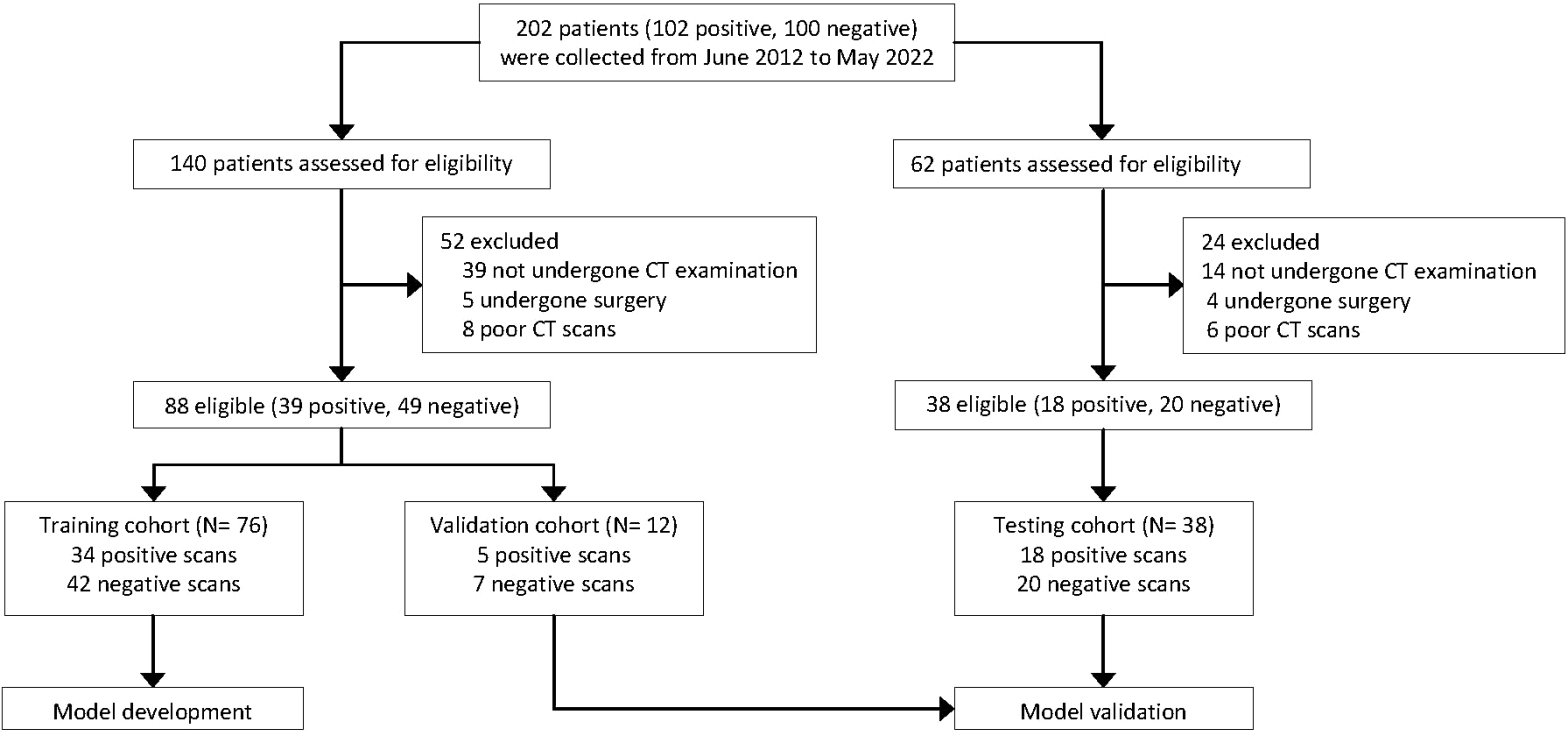
Flow chart showing the overall study process. All computed tomography (CT) scans were retrospectively collected from the clinical databases of a single institution.

We collected 102 patients with pathologically confirmed primary LC who were confirmed to have synchronous or metachronous bone metastases and 100 patients who were confirmed to not have bone metastases by CT-guided biopsy pathology from June 2012 to May 2022. After reviewing the clinical and imaging data, we excluded patients who did not undergo CT examination (n = 53); who underwent surgery, chemotherapy, and radiation therapy for bone metastases before CT examination (n = 9); and who had poor CT image quality (n = 14). Finally, the CT images from 126 patients were included for the DCNN model development to detect bone metastases from LC, including a positive sample dataset of patients with biopsy-proven LC and bone metastases (n = 57), and a negative sample dataset of patients with biopsy-proven LC without bone metastases (n = 69). The process of patient enrollment is shown in **Figure 1**.

We randomly split the whole dataset (n = 126) into three cohorts: training (76 cases, to train the DCNN model), internal validation (12 cases, to fine-tune the hyper-parameters of the DCNN model), and external testing (38 cases, to evaluate the model and radiologists’ performance). Furthermore, the patients included in the validation and testing datasets were excluded from the training dataset.

### 2.2 Imaging preprocessing and annotation

The clinical and imaging information of all patients was obtained through the medical record system and follow-up. To protect patients’ privacy, all identifying information, such as name, sex, age, and ID, on CT was anonymized and omitted through image processing when data were first acquired. After image preprocessing in the Digital Imaging and Communications in Medicine (DICOM) format, complete thin-layer CT images were stored (see Supplementary Methods for CT protocols). The manual annotations of bone metastases were performed using LabelMe (36) with an image segmentation software (Mimics; Materialize, Belgium).

Two board-certified radiologists (8 and 9 years of experience in CT diagnosis) evaluated all CT examinations and, section by section, manually annotated five locations (spine, pelvis, limb, sternum, and clavicle) on the CT images of patients with LCBM. Two expert radiologists (14 and 20 years of experience in CT diagnosis, respectively) checked and manually delineated the volume of interest of the bone metastatic lesion in a voxel-wise manner on CT images using the diagnosis reports from two board-certified radiologists for establishing the reference standard of bone metastases. Furthermore, both of them repeated the annotations and modifications at least 3 weeks later and used them as ground truth (GT) labels for diagnosis and evaluation.

### 2.3 Model development

We developed a cascaded three-dimensional (3D) U-Net with 3D spatial SE modules and 3D GAU modules based on 3D U-Net (37), which is a robust state-of-the-art DCNN-based medical image segmentation method (see Supplementary Methods for training protocol). The Cascaded 3D U-Net (38) contains two 3D U-net architectures, wherein the first one is trained on down-sampled images and the second one is trained on full-resolution images. Training on down-sampled images first can enlarge the size of patches concerning the image and also enable the 3D U-Net network to learn more contextual information. Training on full-resolution images next refines the segmentation results predicted from the former 3D U-Net.

**Figure 2:**
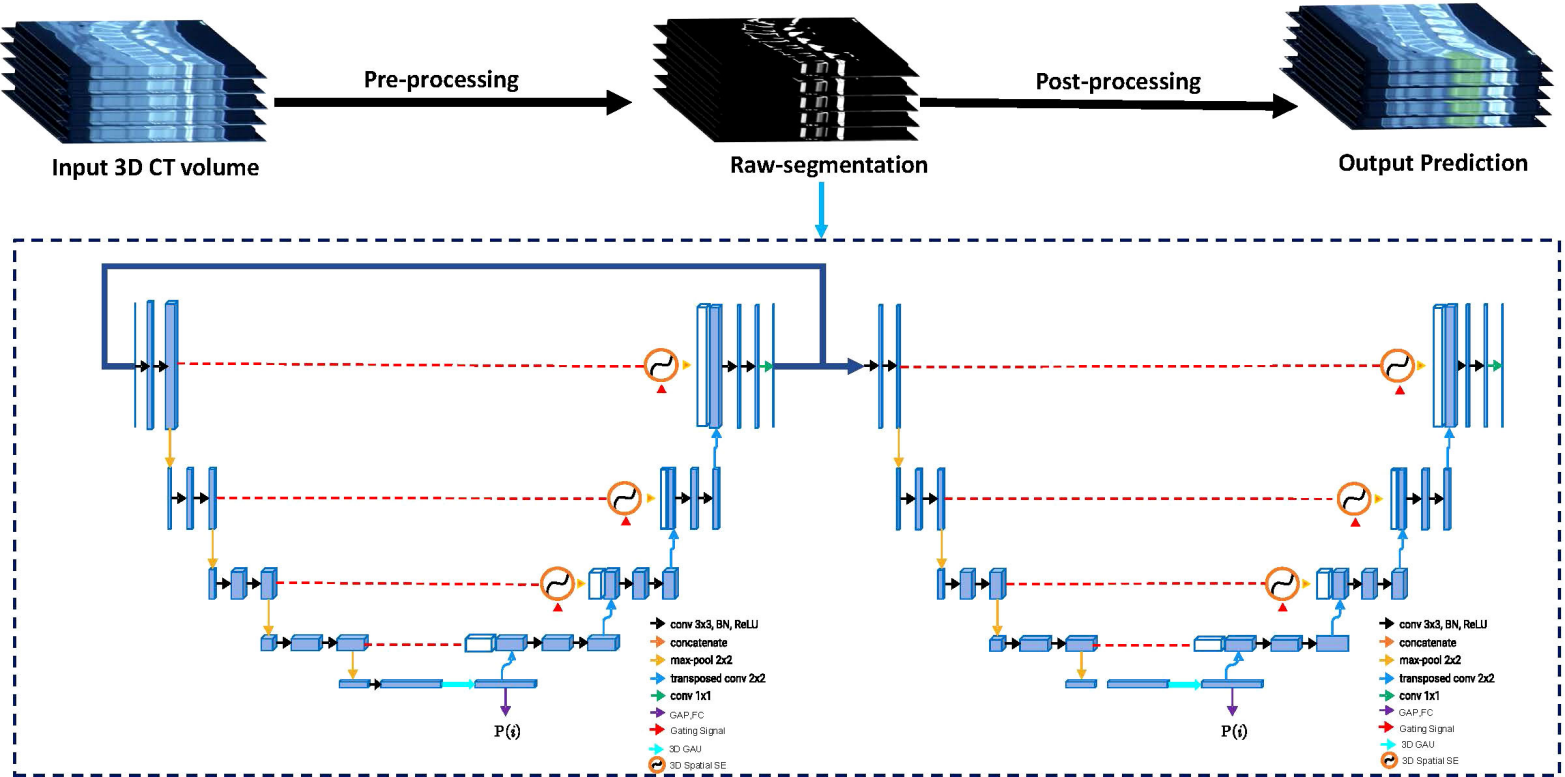
The flowchart of the proposed Cascaded 3D-Unet network. The network structure is divided into two parts: encoder and decoder. Between the encoder and decoder, we used the three-dimensional (3D) spatial squeeze and excitation modules (3D Spatial SE) and 3D global attention-up sample modules (3D GAU) to replace the original skip connections used in 3D U-Net.

The 3D Spatial SE (39) module and 3D GAU (40) module are used more fully as the spatial attention module and the channel attention module to exploit the multiscale and multilevel features, respectively; they guide DCNN to efficiently focus on the targets rather than the background. The flowchart of our DCNN model for segmenting bone metastases is shown in **Figure 2**. The input of the end-to-end DCNN model is the 3D CT volume, and the output is the segmentation result of the bone metastases margin and the possibility of LCBM.

### 2.4 Observer study

To scale the proposed deep learning system with human readers, five radiologists (no overlap with the radiologists who labeled and checked the annotations) with 3–8 years of experience in CT diagnosis were required to participate in an independent human-only observer study. These radiologists were randomly shown the testing dataset to independently segment bone metastases and record the localization (corresponding CT layers); they were blinded to the bone metastases results and patient information.

To simulate the real clinical scenario, a radiologists-DCNN model collaboration study was conducted on three junior radiologists with 1–3 years of CT diagnostic experience, besides the independent observer study. These radiologists independently assessed the images to reach the first conclusion (whether bone metastases are present). Readouts of the DCNN model, including lesion labeling and probability of LCBM, were sent to the radiologists for reevaluating the images. The second assessment of radiologists served as the final output. In addition, we scheduled the 8 radiologists to perform the test in different locations to ensure their relative independence.

### 2.5 Statistical analysis

Our method followed a segmentation methodology to perform a detection task; therefore, both segmentation and detection metrics were important for evaluating the DCNN model performance. The metastases segmentation performance of the network was assessed using the metrics of dice coefficient and Intersection-over-Union (IoU) (41). The dice value indicates the overlapped voxels between the predicted results and GT. Its mathematical definition is as follows:


$$Dice\left(P,\;G\right)=\frac{2\left|P\cap^{\;}G\right|}{\left|P\right|+\left|G\right|}$$

where denotes the number of labeled voxels, and P and G represent the predicted and GT values, respectively. The larger the value of the dice, the higher the degree of overlap between the segmentation prediction and GTs.

The evaluation of detection performance was based on case-based analysis; cases with at least one positive lesion were considered true positive (TP). We compared the model predictions with radiologists with TPs, false negatives (FNs), and false positives (FPs). According to the TP rate (sensitivity) versus the FP rate (1-specificity), we calculated the areas under the receiver operating characteristic (ROC) curve (AUC) and the 95% confidence interval (CI) for the five radiologists (averaged), DCNN model alone, three junior radiologists alone (averaged), and DCNN-assisted three junior radiologists. P< 0.05 was considered statistically significant. All statistical analyses were performed using SPSS 22.0 (IBM Corp., Armonk, NY, USA). The interpretation time for each scan was recorded automatically by the viewer and the detection time of the DCNN model was obtained from the computer terminal.

## 3 Results

No significant difference was noted in age or sex among the monocentric training, validation, and testing cohorts. In total, 34 positive and 42 negative scans were collected for the training dataset. The validation dataset comprised five positive and seven negative scans, and the testing dataset comprised 18 positive and 20 negative scans. The characteristics of the training, validation, and testing cohorts are listed in **Table 1**.

**Table 1:** Characteristics of patients in the three datasets.

Variables	Training Cohort (N=76)	Validation Cohort (N=12)	Testing Cohort (N=38)	Total (N=126)	P value
Age (years), mean ± SD	61.55 ± 13.22	61.48 ± 13.14	61.41 ± 13.45	61.52 ± 13.25	<0.001
Gender					<0.001
Male	44 (57.8%)	7 (58.3%)	22 (57.9%)	73 (57.9%)	
Female	32 (42.2%)	5 (41.7%)	16 (42.1%)	53 (42.1%)	
Positive with bone metastases	33 (43.4%)	5 (41.7%)	19 (50.0%)	57 (45.2%)	
Bone metastasis site					0.417
Spine	14 (42.4%)	2 (40.0%)	8 (42.1%)	24 (42.1%)	
Pelvis	10 (30.3%)	1 (20.0%)	6 (31.6%)	17 (29.8%)	
Limb	5 (15.2%)	1 (20.0%)	3 (15.8%)	9 (15.8%)	
Sternum	3 (9.1%)	1 (20.0%)	2 (10.5%)	6 (10.5%)	
Clavicle	1 (3.0%)	0 (0.0%)	0 (0.0%)	1 (1.8%)	
Negative without bone metastases	43 (56.6%)	7 (58.3%)	19 (50.0%)	69 (54.8%)	
Brain metastases	9 (20.9%)	2 (28.6%)	4 (21.1%)	15 (21.7%)	
Liver metastases	26 (60.5%)	4 (57.1%)	12 (63.2%)	42 (60.9%)	
Lymph nodes metastases	8 (18.6%)	1 (14.3%)	3 (15.8%)	12 (17.4%)	
Primary Subtype					0.034
Small cell lung cancer (SCLC)	63 (82.9%)	10 (83.3%)	32 (84.2%)	105 (83.3%)	
Adenocarcinoma (LUAD)	13 (17.1%)	2 (16.7%)	6 (15.8%)	21 (16.7%)	
Number of tumors					0.002
Single	59 (77.6%)	9 (77.6%)	31 (81.6%)	99 (78.8%)	
Multiple (2)	17 (22.4%)	3 (22.4%)	8 (18.4%)	27 (21.4%)	
Tumor size					0.124
0≤X ≤ 3	16 (20.5%)	2 (16.7%)	8 (21.1%)	26 (20.3%)	
3<X ≤ 5	25 (32.9%)	3 (25.0%)	13 (34.2%)	41 (32.5%)	
5<X ≤ 7	17 (22.4%)	4 (33.3%)	9 (23.7%)	30 (23.8%)	
>7	18 (23.7%)	3 (25.0%)	8 (21.1%)	29 (23.0%)	
Grade					0.297
Grade I	4 (5.3%)	3 (25.0%)	9 (23.7%)	31 (24.6%)	
Grade II	19 (25.0%)	8 (66.7%)	23 (60.5%)	75 (59.5%)	
Grade III	44 (57.9%)	1 (8.3%)	4 (10.5%)	14 (11.1%)	
Grade IV	9 (11.8%)	3 (25.0%)	9 (23.7%)	31 (24.6%)	

For patient age, the mean age and standard deviation are presented, with a range of values in parentheses. For other data, the number of patients is presented, with percentages in parentheses.

### 3.1 Performance of the DCNN model

**Figure 3:**
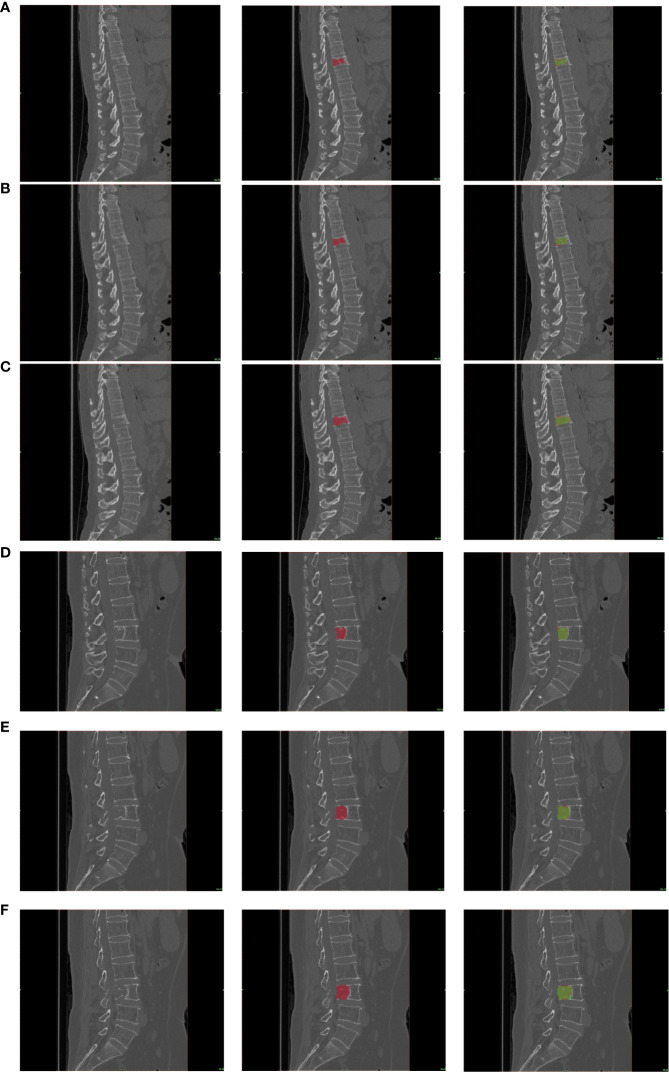
The segmentation results. Representative example of a patient with lung cancer bone metastasis with abnormal signals in the 4 lumbar spine **(A–C)** and a patient with lung cancer bone metastasis with abnormal signals in the 11 thoracic spine **(D–F)**, and true-positive lesions with various appearances and locations. From left to right, the three images in a row are the original image, the corresponding GT label (red), and the candidate region output from the deep convolutional neural network (DCNN) (green). Note that the green region on the right image is a candidate region with a threshold of 0.5, and dice was calculated on this region.

A threshold of 0.5 (IoU > 0.5) was defined as the detection hit criterion; then, the segmentation metrics were computed with the GT labels generated in the image annotation procedure. At a threshold of 0.5, the detection sensitivity of our DCNN model was 0.898 with 5.23 average FPs for the validation dataset and 0.894 with 5.24 average FPs for the testing dataset. Besides, our DCNN model achieved an acceptable segmentation performance (dice = 0.859 and 0.856 on the validation and testing datasets, respectively). The overall results of the DCNN model for the validation and testing datasets are shown in **Table 2**. An illustration of the predicted segmentation by DCNN is shown in **Figure 3**, where all cases were predicted with highly similar segmentation to GT.

**Table 2:** Results of comparison with other networks and five radiologists on the testing cohort.

	AUROC (95% CI)	Accuracy (95% CI)	Sensitivity (95% CI)	Specificity (95% CI)	IoU	Dice	Avg FP
Validation Cohort							
3D FCN	0.775 (0.746–0.803)	0.777 (0.724–0.823)	0.811 (0.792–0.840)	0.741 (0.720–0.766)	0.674 (0.643–0.750)	0.766 (0.736–0.796)	7.64
3D U-NET	0.781 (0.746–0.810)	0.790 (0.738–0.836)	0.810 (0.790–0.843)	0.752 (0.741–0.779)	0.681 (0.686–0.791)	0.785 (0.759–0.819)	7.20
3D GAU U-Net	0.805 (0.798–0.832)	0.807 (0.787–0.821)	0.784 (0.760–0.806)	0.834 (0.809–0.859)	0.693 (0.680–0.722)	0.794 (0.758–0.828)	5.97
3D SSE U-Net	0.853 (0.845–0.881)	0.853 (0.838–0.868)	0.860 (0.795–0.889)	0.850 (0.825–0.873)	0.750 (0.721–0.784)	0.827 (0.781–0.859)	5.65
Cascaded 3D U-Net	0.871 (0.849–0.880)	0.877 (0.821–0.907)	0.893 (0.881–0.905)	0.866 (0.835–0.875)	0.751 (0.701–0.790)	0.843 (0.812–0.888)	5.41
**Our network**	**0.883 (0.878–0.901)**	**0.886 (0.842–0.922)**	**0.898 (0.881–0.905)**	**0.863 (0.835–0.875)**	**0.782 (0.741–0.810)**	**0.859 (0.826–0.884)**	**5.23**
Testing Cohort							
3D FCN	0.771 (0.749–0.784)	0.773 (0.751–0.794)	0.806 (0.789–0.822)	0.736 (0.710–0.759)	0.677 (0.651–0.754)	0.762 (0.732–0.790)	7.74
3D U-NET	0.775 (0.756–0.796)	0.784 (0.787–0.801)	0.802 (0.785–0.818)	0.748 (0.723–0.770)	0.680 (0.664–0.782)	0.781 (0.750–0.818)	7.50
3D GAU U-Net	0.803 (0.776–0.834)	0.810 (0.799–0.853)	0.778 (0.761–0.818)	0.817 (0.801–0.844)	0.691 (0.674–0.718)	0.791 (0.764–0.823)	6.12
3D SSE U-Net	0.846 (0.828–0.867)	0.847 (0.783–0.897)	0.857 (0.786–0.891)	0.847 (0.819–0.868)	0.749 (0.720–0.798)	0.822 (0.779–0.855)	5.74
Cascaded 3D U-Net	0.868 (0.838–0.872)	0.875 (0.834–0.908)	0.887 (0.881–0.905)	0.854 (0.835–0.867)	0.750 (0.706–0.783)	0.841 (0.810–0.888)	5.58
**Our network**	**0.875 (0.863–0.883)**	**0.878 (0.867–0.886)**	**0.894 (0.874–0.896)**	**0.857 (0.831–0.885)**	**0.789 (0.733–0.808)**	**0.856 (0.820–0.885)**	**5.24**
Radiologist 1	0.785 (0.759–0.800)	0.771 (0.755–0.798)	0.846 (0.797–0.887)	0.766 (0.737–0.783)	0.642 (0.611–0.688)	0.726 (0.698–0.750)	3.25
Radiologist 2	0.792 (0.776–0.822)	0.793 (0.768–0.802)	0.861 (0.813–0.900)	0.769 (0.733–0.787)	0.668 (0.639–0.690)	0.741 (0.700–0.777)	3.10
Radiologist 3	0.795 (0.779–0.799)	0.799 (0.773–0.813)	0.876 (0.830–0.913)	0.776 (0.753–0.796)	0.679 (0.652–0.721)	0.750 (0.712–0.789)	2.68
Radiologist 4	0.804 (0.791–0.825)	0.810 (0.795–0.827)	0.880 (0.875–0.925)	0.792 (0.770–0.819)	0.691 (0.670–0.744)	0.769 (0.722–0.808)	2.14
Radiologist 5	0.819 (0.800–0.849)	0.822 (0.801–0.839)	0.892 (0.891–0.929)	0.807 (0.792–0.831)	0.717 (0.700–0.762)	0.798 (0.759–0.841)	1.80
Average of five radiologists	0.799 (0.772–0.821)	0.799 (0.782–0.834)	0.871 (0.841–0.899)	0.782 (0.758–0.800)	0.679 (0.654–0.702)	0.757 (0.730–0.783)	2.59

A comparison of detection and segmentation performance in the testing dataset of our proposed network (Cascaded 3D U-Net with 3D GAU modules and the 3D SSE modules), five other deep neural networks [Cascaded 3D U-Net, 3D FCN, 3D U-net, 3D GAU U-Net (3D U-Net with 3D global attention-up sample modules, and 3D SSE U-Net (3D U-Net with spatial squeeze and excitation modules)]. FP: false positives per scan. IoU: Intersection-over-Union. Dice: dice coefficient The bolded words “Our network” represent our proposed network (Cascaded 3D U-Net with 3D GAU modules and the 3D SSE modules). The bolded words “Validation Cohort” and “Testing Cohort” represent two equal cohorts containing different data.

### 3.2 Comparison with other networks and radiologists

**Figure 4:**
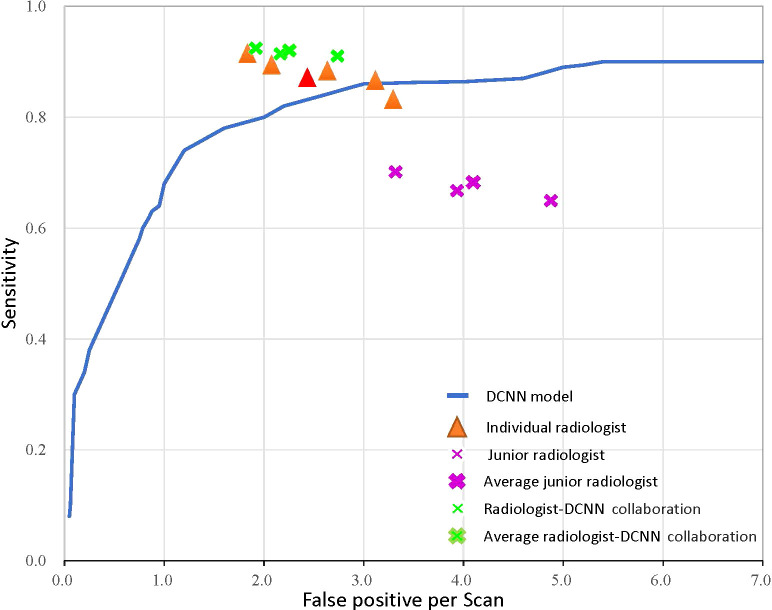
The receiver operator characteristic curve for the performance of the deep convolutional neural network (DCNN) model-only, radiologist-only, junior radiologist-only, and junior radiologists-DCNN model collaboration detection performance. Each orange triangle represents the performance of an individual radiologist; each pink fork represents the performance of a junior radiologist without the aid of DCNN; and each green fork represents the performance of a junior radiologist with the aid of DCNN. The red triangle indicates the average value of five radiologists, and the bolded forks indicate the average value of junior radiologists.

We compared our model with several state-of-the-art deep neural networks in the validation and testing datasets to validate the effectiveness of the proposed DCNN model (**Figure 4** and **Table 2**). As shown in the results, the Cascaded 3D U-Net outperformed the 3D U-NET and 3D FCN (42) by large margins, which verified the effectiveness of network design in the proposed DCNN model. Moreover, the results of the ablation experiments demonstrated the best performance of our 3D U-Net with a 3D GAU module and 3D spatial SE module.

We performed observer studies and compared them with five radiologists using all images in the testing dataset to characterize the diagnostic value of the DCNN model. The DCNN model achieved high performance, outperforming any radiologists for LCBM detection with respect to both the primary metric AUROC (0.875 vs. 0.819 for the best radiologist) and sensitivity (0.894 vs. 0.892) tested in the observer-independent study. As for segmentation performance, our DCNN model outperformed all other networks and five radiologists (**Table 2**).

### 3.3 Radiologists-DCNN model collaboration

We further validated the radiologists-DCNN model collaboration performance in the testing dataset. As demonstrated, the three junior radiologists (with the assistance of the DCNN model) showed substantial improvement in identifying LCBM (**Figure 4** and **Table 3**).

**Table 3:** A comparison of the DCNN model and three junior radiologists without and with the DCNN model.

		Age (year)	Experiences (year)	AUROC (95% CI)	Accuracy (95% CI)	Sensitivity (95% CI)	IoU	Dice	Avg Time (s)	Avg FP
**DCNN Model**		**–**	**–**	**0.875 (0.863–0.883)**	**0.878 (0.867–0.886)**	**0.894 (0.874–0.896)**	**0.789 (0.733–0.808)**	**0.856 (0.820–0.885)**	**27**	**5.24**
Junior radiologist only	R6	25	1.5	0.584 (0.567–0.612)	0.597 (0.575–0.614)	0.657 (0.531–0.768)	0.498 (0.480–0.515)	0.589 (0.568–0.610)	412	4.89
	R7	31	2	0.601 (0.588–0.620)	0.609 (0.590–0.632)	0.672 (0.546–0.782)	0.576 (0.534–0.604)	0.663 (0.638–0.686)	312	3.95
	R8	34	3	0.643 (0.611–0.668)	0.645 (0.622–0.671)	0.711 (0.695–0.741)	0.620 (0.586–0.645)	0.708 (0.684–0.733)	387	3.36
	**Avg**	**30**	**2.17**	**0.609 (0.591–0.634)**	**0.617 (0.602–0.640)**	**0.680 (0.543–0.753)**	**0.565 (0.543–0.598)**	**0.653 (0.637–0.698)**	**370.3**	**4.07**
Junior radiologist with DCNN	R6	25	1.5	0.871 (0.852–0.896)	0.875 (0.860–0.900)	0.898 (0.870–0.922)	0.771 (0.748–0.794)	0.833 (0.800–0.867)	204	2.72
	R7	31	2	0.873 (0.856–0.891)	0.879 (0.875–0.911)	0.901 (0.864–0.924)	0.790 (0.771–0.827)	0.861 (0.811–0.893)	104	2.23
	R8	34	3	0.878 (0.861–0.901)	0.883 (0.869–0.923)	0.907 (0.854–0.924)	0.802 (0.784–0.821)	0.886 (0.844–0.928)	119	1.98
	**Avg**	**30**	**2.17**	**0.874 (0.857–0.914)**	**0.879 (0.863–0.910)**	**0.902 (0.844–0.921)**	**0.788 (0.764–0.823)**	**0.847 (0.812–0.869)**	**142.3**	**2.31**

FP, false positives per scan. IoU, Intersection-over-Union. Dice, dice coefficient. The bolded words “DCNN Model” represent our proposed network (Cascaded 3D U-Net with 3D GAU modules and the 3D SSE modules). The bolded words “Avg” represent the average of the values in the three cells above.

The DCNN model assisted junior radiologists in diagnosing LCBM with a higher mean AUROC (0.874 [95% CI: 0.807–0.874] vs. 0.609 [95% CI: 0.591–0.634], P< 0.001), mean accuracy (0.879 vs. 0.617, P< 0.001), and mean sensitivity (0.902 vs. 0.680, P = 0.009) compared with those achieved alone. Moreover, the mean interpretation time per case of the junior radiologists was significantly reduced from 370.3 s to 142.3 s (228 s decrease, P = 0.045) when assisted by the DCNN model.

## 4 Discussion

Herein, we proposed an improved Cascaded 3D U-Net based on the DCNN model to detect and segment LCBM on CT scans. Radiologists underperformed the DCNN model concerning detection sensitivities, although they achieved much lower average FPs. In the segmentation task, the proposed DCNN model achieved a mean dice of 0.856 and a mean FP of 5.24 in the testing dataset, showing that the proposed model achieved better results than other state-of-the-art networks.

In the radiologists-DCNN model collaboration study, the mean sensitivity of the junior radiologist for LCBM improved from 0.680 to 0.902 with acceptable FPs (2.59). The radiologists-DCNN model collaboration enhanced the detection sensitivity and FPs compared with radiologist-only or DCNN model-only diagnosis, demonstrating the existence of the DCNN model-detected bone metastases that were missed by junior radiologists and vice versa. Moreover, the DCNN model-assisted diagnosis significantly decreased approximately 62% clinical time (142 s vs. 370 s), which had never been evaluated in previous studies.

Prior to our study, two recent studies used deep learning to detect bone metastases from CT images (43, 18). However, both studies focused only on spinal lesions. The spine is the most common site of bone metastases; however, metastases can occur at any site in the entire skeleton (44). Furthermore, both studies formalized the task as two-dimensional detection, whereas our study formalized it as 3D segmentation. Besides, the data and annotation in our study were of a higher standard. In our study, high-quality thin-slice CT scans with a thickness of 1–1.25 mm were used to support the model development. Moreover, we followed a repetitive and retrospective labeling procedure by four radiologists to ensure the high quality of our annotations, thus reducing the risk of overvaluing model performance. Our model achieved significantly higher detection sensitivity and remained consistent across the training, validation, and testing datasets.

Nevertheless, there are still some limitations to this study. First, our study was retrospective and monocentric; therefore, future validations in prospective randomized settings can provide more powerful conclusions. In addition, the sample size was small. We believe that the main reason for such limitation is the somewhat low incidence and prevalence of LCBM (45). Although our results were encouraging, experiments in large multi-center datasets are needed to verify the results in further studies. Second, the training process of the DCNN model depends on the segmentation GT labels of LCBM by radiologists, who also have imperfect reliability. To address this issue, numerous studies have been conducted to train the DCNN model using weak labels (46). Third, the DCNN model was established to detect LCBM so that the patients included in the positive cohort had LC. However, bone metastases may also arise from other solid tumors, such as breast, prostate, colorectal, thyroid, and gynecologic cancers and melanoma (47). Therefore, more generalized DCNN models that can distinguish multiple origins of bone metastases should be followed up. Finally, our DCNN models were designed to deal with a single task of LCBM detection on CT. However, in clinical practice, radiologists may not rely on a single medical file for a final diagnosis, instead, they need to combine other clinical or imaging reports to achieve the diagnosis of LCBM. If the models are built based on a single parameter, their clinical value may be significantly endangered. Therefore, more inclusive models combining various characteristics should be designed and emphasized in the future (48).

In conclusion, our DCNN model collaborated with junior radiologists helped to enhance the diagnostic effectiveness and efficiency in the diagnosis of LCBM on CT, indicating the great potential of DCNN-assisted diagnosis in clinical practice.

## Data availability statement

The raw data supporting the conclusions of this article will be made available by the authors, without undue reservation.

## Ethics statement

This retrospective study was approved by the hospital ethics committee (Union Hospital, Tongji Medical College, Huazhong University of Science and Technology). The ethics committee waived the requirement of written informed consent for participation.

## Author contributions

TH and YX contributed equally to the manuscript. ZY and SZ present the conception of the work; YF and MX acquired the data; SL obtained funding to support the work and built up the research team for joint development; YD and LL labelled the images for further analysis; JZ and HL assisted in collecting data. TH, ZW and PL conducted the statistical analysis; TH drafted the manuscript with critical feedback from all authors. All authors contributed to the article and approved the submitted version.
