# Supplementary material for: Deep learning-based algorithm improves radiologists’ performance in lung cancer bone metastases detection on computed tomography
Source: Front Oncol. 2023 Feb 8;13:1125637. doi: 10.3389/fonc.2023.1125637 (PMC9946454; doi:10.3389/fonc.2023.1125637)
Supplement: Supplementary file 1 [file DataSheet_1.docx]

Supplementary Material

# Supplementary Methods

## Computed tomography (CT) protocols

All the CT volume scans were primarily performed using three scanners (Brilliance 16, Philips Healthcare, Eindhoven, the Netherlands; Ingenuity 128, Philips Healthcare, Cleveland, OH, USA; Somatom Definition Flash, Siemens Healthineers, Forchheim, Germany). The scanning parameters were as follows: 120 kV; 100–200 mA; pitch, 0.75–1.5; collimation, 1–1.25 mm; and matrix, 512×512 (600 slices on average), respectively. A bone reconstruction algorithm was used with a reconstructed slice thickness was 1, 2, or 5 mm.

## Pre-processing

To accelerate the detection, we extracted the bone areas through a series of morphological operations (e.g., thresholding and filtering). The original spacing was preserved since only thin-section CT scans were included in our dataset. The intensity of input voxels was clipped to the bone window (level = 450, width = 1100) and normalized to [−1, 1]. Since a whole-volume CT scan could be too large to fit in a regular GPU memory, we cropped 64 × 64 × 64 patches in a sliding-window fashion with a stride of 48 and fed them into our network. A raw segmentation was obtained by assembling patches of prediction. Maximum values were kept in the overlapping regions of multiple predictions. A variety of data augmentation techniques are applied on the fly during training: rotations, scaling, Gaussian noise, Gaussian blur, brightness, contrast, simulation of low resolution, gamma correction and mirroring.

## Post-processing

Connected component-based post-processing is commonly used in medical image segmentation. Especially in organ image segmentation, it often helps to eliminate the detection of spurious false positives by removing all but the largest connected component. Our 3D U-Net model follows this assumption and automatically benchmarks the effect of suppressing smaller components on the cross-validation results. First, all foreground classes are treated as one component. If suppression of all but the largest region improves the average foreground dice coefficient and does not reduce the dice coefficient for any of the classes, this procedure is selected as the first post-processing step. Finally, our model builds on the outcome of this step and decides whether the same procedure should be performed for individual classes.

## Training protocol

In this study, we used the improved Cascaded 3D U-Net method to segment lung cancer bone metastases (LCBM) on CT images. Automatic medical image segmentation plays a significant role as a diagnostic aid in the identification of diseases and their treatment in clinical settings, thus requiring lesser time and experience than manual manipulation. Among the deep convolutional neural network (DCNN) models, the U-Net is a well-known model that was proposed for the segmentation of biomedical images and has been shown to work well on few-shot datasets. The 3D U-Net architecture comprises an encoder and a decoder that extract low-level features and high-level features, respectively. The encoder extracts the features by a series of convolution and max-pooling layers, whereas the decoder recovers image resolution by deconvolution layers. However, the levels of features in the encoder path are much lower than those in the decoder path; therefore, it is not favorable enough to make full use of the multiscale and multilevel features by simply using the skip connections to concatenate the feature maps from different paths. Therefore, we introduced two attention modules to emphasize meaningful features along the spatial and channel axes, respectively. The 3D Spatial SE module and 3D GAU module are used as the spatial and channel attention modules, respectively, and they guide CNN to efficiently focus on the targets rather than the background.

To efficiently reduce the false positive in our predictions, predictions of small sizes (smaller than 200 voxels) were filtered out. To generate a detection proposal, we first binarized the post-processed segmentation results with a low threshold of 0.1 and then computed connected components on the binary segmentation. Each connected component was regarded as a detection proposal, with a probability calculated by averaging raw segmentation scores over all voxels within the connected component. To equalize the unbalanced dataset, we assigned different weights to each class when computing the loss. Based on experience and as a trade-off between runtime and reward, all networks are trained for 1,000 epochs, with one epoch being defined as iteration over 250 mini-batches. The training optimizer used stochastic gradient descent (SGD) with a momentum setting of 0.9, and the learning rate strategy used phase decay with an initial learning rate of 0.01. The learning rate decreased to 1/10 of the original rate every 7 epochs. The loss function is the sum of cross-entropy and dice loss. For each deep supervision output, a corresponding down sampled ground truth segmentation mask is used for loss computation. Samples for the mini-batches are chosen from random training cases. Oversampling is implemented to ensure robust handling of class imbalances. The number of foreground patches is rounded with a forced minimum of 1 (resulting in one random and one foreground patch with a batch size of two).

The DCNN model outputs multiple boxes when predicting a target; for example, there are various boxes corresponding to the same target. When the IoU≥ threshold, it is considered a true positive prediction box (TP: True-Positive). In contrast, if the IoU<threshold, it is considered a false positive prediction (FP: False-Positive). Calculating the precision [precision=TP/(TP+FP)] and recall [recall=TP⁄(TP+FN)] of positive samples, we obtain the points on each P-R curve (with recall as the horizontal coordinate and precision as the vertical coordinate). Then, we fixed the IoU threshold and changed the confidence threshold (0: 1: 0.1) to obtain the other points on the P-R curve. All points were connected to obtain the complete P-R curve, and the AP was calculated from the area under the P-R curve. The algorithm outputs different results according to the preset threshold; thus, the results for each threshold from 0.1 to 0.9 are presented. The number of FP per case increased with increasing the threshold. Based on the results for the validation dataset, a threshold of 0.5 was defined as the standard value, since too many FP outputs may impede clinical assessments. Furthermore, according to the model comparison results, Cascaded 3D U-Net has a significant advantage over other detection models when IOU=0.5. In other words, Cascaded 3D U-Net is faster and more accurate than the other models at this time. All training and testing procedures was developed with PaddlePaddle2.0.2, CUDA10.1 and Python 3.7. Four graphics processing units (GPUs) (NVIDIA GeForce GTX 1080Ti) were used, and the total training time was 10 days.
